# Malaria in Senegal: Recent and Future Changes Based on Bias-Corrected CMIP6 Simulations

**DOI:** 10.3390/tropicalmed7110345

**Published:** 2022-11-01

**Authors:** Ibrahima Diouf, Jacques-André Ndione, Amadou Thierno Gaye

**Affiliations:** 1 Laboratoire de Physique de l’Atmosphère et de l’Océan-Siméon Fongang, Ecole Supérieure Polytechnique de l’Université Cheikh Anta Diop (UCAD), BP 5085, Dakar-Fann, Dakar 10700, Senegal; 2 Regional Agency for Agriculture and Food, Lomé 01 BP 4817, Togo

**Keywords:** projections, CMIP6, climate, impacts, health, malaria, Senegal

## Abstract

Malaria is a constant reminder of the climate change impacts on health. Many studies have investigated the influence of climatic parameters on aspects of malaria transmission. Climate conditions can modulate malaria transmission through increased temperature, which reduces the duration of the parasite’s reproductive cycle inside the mosquito. The rainfall intensity and frequency modulate the mosquito population’s development intensity. In this study, the Liverpool Malaria Model (LMM) was used to simulate the spatiotemporal variation of malaria incidence in Senegal. The simulations were based on the WATCH Forcing Data applied to ERA-Interim data (WFDEI) used as a point of reference, and the biased-corrected CMIP6 model data, separating historical simulations and future projections for three Shared Socio-economic Pathways scenarios (SSP126, SSP245, and SSP585). Our results highlight a strong increase in temperatures, especially within eastern Senegal under the SSP245 but more notably for the SSP585 scenario. The ability of the LMM model to simulate the seasonality of malaria incidence was assessed for the historical simulations. The model revealed a period of high malaria transmission between September and November with a maximum reached in October, and malaria results for historical and future trends revealed how malaria transmission will change. Results indicate a decrease in malaria incidence in certain regions of the country for the far future and the extreme scenario. This study is important for the planning, prioritization, and implementation of malaria control activities in Senegal.

## 1. Introduction

The threat of climate change is well known, and it has a huge impact on the emergence and re-emergence of certain vector-borne infectious diseases such as malaria. This disease continues to evolve in a changing world, and its geographical distributions are due to changes in vector transmission, which is influenced by rising temperature, moisture availability, and rainfall [1,2]. The climate change impact on health will not be evenly distributed, and some health burdens will be opposite to the global distribution of greenhouse gas emissions [3]. The Intergovernmental Panel on Climate Change (IPCC) has concluded that to avert catastrophic health impacts and reduce the projected millions upon millions of climate change-related deaths, the world must avoid or even limit the increase in temperature and other inevitable changes. Even at 1.5 °C, global warming is not to be considered insignificant because every tenth of a degree in additional warming will impact the lives and health of populations [4]. Developing effective and sustainable human health services, associated surveillance and emergency preparedness systems, and disease control and prevention can help mitigate climate change’s effects on human health. The WHO Global Technical Strategy (GTS) for malaria sets a goal to reduce case incidence and malaria deaths by 90% by the year 2030 relative to 2015 levels [5]. These targets have been missed by 37% and 22%, respectively, because malaria-determining factors such as climate information, land use/cover change, resistance (parasite, insecticide), accurate diagnosis, socioeconomic status, and other related environmental factors are not operationally used in health decision-making processes. Malaria is now well documented by scientific work, not only in the context of diseases, but above all, by work related to variability and/or climate change. Our study in this paper focuses on evaluating the past and current impact of seasonal and interannual climate variability on malaria in Senegal. Malaria is a vector-borne disease whose existence and transmission depend on three main factors: the *Plasmodium* spp. parasite, the *Anopheles* vector, and the human host. Beyond these essential factors, the risk of malaria transmission can be affected by environmental conditions or climatic conditions, as well as socio-economic factors. In particular, the transmission of malaria is very sensitive to climate and atmospheric conditions. When these are unusual, for example, during heavy rainfall, mosquito populations can multiply and trigger epidemics. We have previously studied the climate–malaria relationship [6] as well as the predictability of high malaria occurrences in Senegal and West Africa in general [7]. These studies have already noted a causal relationship between El Niño and malaria parameters. These results were endorsed by Diouf et al. [8] by coupling the Liverpool Malaria Model (LMM) of Hoshen and Morse [9] and the SST-based statistical ForeCAST (S4CAST) of Suárez-Moreno and Rodríguez-Fonseca et al. [10]. In these previous studies, firstly, using daily rainfall and daily 2 m temperature of available reanalysis datasets, we applied the Liverpool Malaria Model (LMM) to simulate malaria indices and validated our simulations with malaria surveillance data from the National Malaria Control Programme (Programme National de Lutte contre le Paludisme, PNLP) in Senegal. Secondly, we focused on the predictability of malaria outbreaks in our area of study. Indeed, climate forecasts at a sufficient lead time are needed to allow response planning from users in several sectors including health, and sea surface temperatures (SSTs) are the main variables that can be used for this aim. Temperature almost instantaneously affects malaria outbreaks, while the impact of rainfall on malaria transmission is usually noted with a 1-month to 2-month lead time [6,11]. SST is a variable that reflects the anomalous energy storage in the ocean, due to the thermal inertia of the ocean. This anomalous energy is released in the forthcoming months, altering atmospheric variability, and impacting climate-dependent vector-borne diseases such as malaria. The justification for how this present analysis differs from previous studies in Senegal rests on the climate change impacts on malaria using bias-corrected reanalysis data such as the WFDEI data as a point of reference and bias-corrected CMP6 simulations and the use of the latest scenarios, so-called Shared Socioeconomic Pathways scenarios (SSPs). In addition, the novelty of this study includes the investigation of the long-term changes and reliability of projections as driving conditions for the malaria model, separating historical data and projections for three SSPs (SSP126, SSP245, and SSP585) and using pieces of evidence from the published literature.

This work is organized as follows. Section 2 presents the different types of data used and the methods applied. Section 3 studies the spatiotemporal variability of the health and climatic data (rainfall and temperature) over Senegal, and the malaria simulation variable with the LMM model forced by the previously presented data. Finally, a summary and discussion of the main results are provided in Section 4.

## 2. Materials and Methods

### 2.1. Study Area

**Figure 1:**
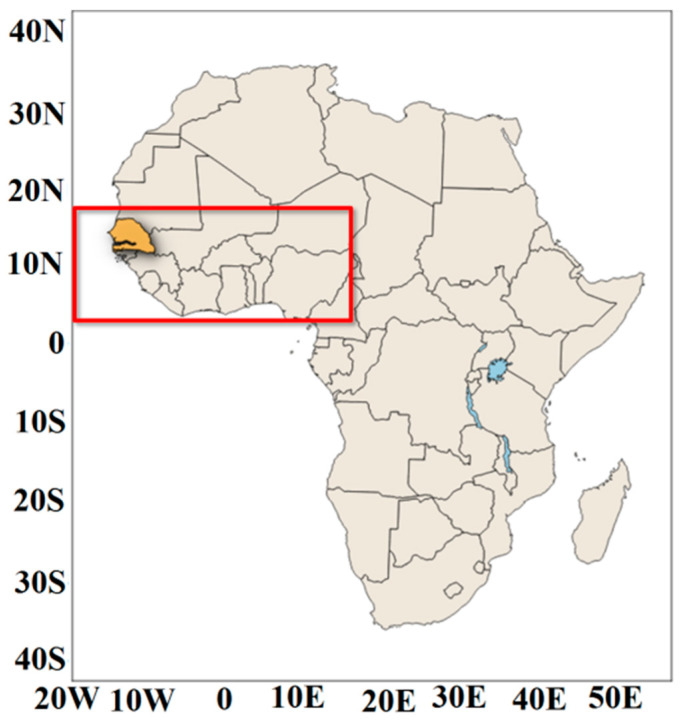
Location of the study area: Senegal (12 to 17 N and 18 to 11 W) is colored in yellow, located in West Africa (4 to 18 N and 20 W to 15 E), which is delineated in red.

Senegal is located at the westernmost tip of West Africa and lies within 12–17° N latitude and 18–12° W longitude (Figure 1). The description based on the practices of African meteorologists who meet the Köppens classification [12] indicates that climate characteristics are very diverse across different ecoregions of West Africa, ranging from a Sahelian to a Sudano-Sahelian and a Sudano-Guinean climate. Toward the north of West Africa, including Senegal, the climate is Sahelian, with moderate rainfall occurring during a short rainy season (August–September). Senegalese climate is strongly modulated by the West African monsoon. The temperature in Senegal presents a bimodal evolution with two peaks in May and October, on either side of the rains. The climate of Senegal is characterized by great spatiotemporal variability, and its dynamics are generally dominated by a northeast wind regime (Harmattan) during the dry season and southwest regime during the rainy season (West African Monsoon). The period from December to February is the coldest, but the continental influence is more clearly visible in the east on the diurnal thermal difference. May and October are generally the hottest months for most of the country. For the rainfall, a latitudinal gradient is generally observed; the amount of rainfall decreases along the south–north direction due to the arrival of the West African Monsoon (WAM), which moves into the country through the southern part of Senegal. The first significant rains begin between June–July and are given by squall lines whose effectiveness decreases towards the west [13]. The rains continue until October. Cumulative rainfall varies from 300 mm to 1000 mm [14].

Regarding climate change, like many Sahelian countries, Senegal faces many natural hazards such as floods, droughts, and heat waves. The latter are extreme weather events generally characterized by the persistence of high temperature for several days. With the progression of climate change, these heat waves are expected to increase in number and intensity and everywhere in the world [15].

### 2.2. Data Used

#### 2.2.1. Malaria Data Surveillance

Regarding observation, we acquired malaria data from health districts, from MSAS (Ministère de la Santé et de l’Action Sociale; Ministry of Health and Social Action/MHSA) sentinel sites. The health data obtained so far contain the number of disease cases recorded on an annual scale and by region. The clinical data corresponding to the number of observed malaria cases were obtained from the PNLP (Programme National de Lutte contre le Paludisme au Senegal; NMCP/National Malaria Control Programme in Senegal in English). The PNLP records malaria cases in the framework field surveys in Senegal. The observed numbers of malaria cases used in this study were collected from various health districts in Senegal for the period 2011–2021. These malaria data were recorded for all health districts and hospitals to derive a monthly time series for 14 administrative regions (Dakar, Diourbel, Fatick, Kaffrine, Kaolack, Kedougou, Kolda, Louga, Matam, Saint-Louis, Sedhiou, Tambacounda, Thies, and Ziguinchor). These sentinel sites provide a good representation of malaria transmission in the different climatic zones of Senegal.

#### 2.2.2. Climate Data and Methods

In this study, we used climate data from the WATCH Forcing Data applied to ERA-Interim data (WFDEI) as reference data [16]. The multi-model ensemble mean of fifteen (15) global circulation models included, for instance, the Coupled Model Intercomparison Project, version 6, where a bias correction technique for the climate variables including rainfall and temperature, the CDF-t (Cumulative Distribution Function transform) method [17], was applied. The WATCH Forcing Data methodology applied to ERA-Interim data (WFDEI) [18] is produced from Watch Forcing Data (WFD) and ERA-Interim re-analyses via sequential interpolation at 0.5° resolution, an altitude correction and monthly scale adjustments based on monthly observational data from CRU (Climate Research Unit) TS3.1/TS3.21 and GPCCv5/v6. Details of the three products can be found in Dee et al. [16] for ERA-Interim and Weedon et al. [18] for WFDEI. The variables have a daily time step with a spatial resolution of 0.75° (~80 km) and 0.50° (~55 km), respectively, for ERA-Interim and WFDEI, being qualified as low resolution and medium resolution.

Regarding the CMIP6, these simulations are available on a daily time scale for the period 1850–2014 (historical) and 2015–2100 (projections). The available models are shown in Table 1. They are available at different spatial resolutions (Table 1), which, for example, range from 50 km (CNRM-CM6_HR) to more than ~300 km (CanESM5). The period 1985 to 2014 was chosen as the reference period.

To study possible future impacts, the Intergovernmental Panel on Climate Change (IPCC) in its sixth report [19] relies on five scenarios called Shared Socio-economic Pathways (SSP). Economists and sociologists assess the costs of adaptation and mitigation related to climate change according to different socio-economic scenarios compatible with the Representative Concentration Pathway (RCP) scenarios. The RCPs are four trajectory scenarios of radiative forcing up to 2100. These scenarios were used in the IPCC Fifth Assessment Report (AR5). In the fifth assessment report of the IPCC (AR5, published in 2013) and based on four different hypotheses concerning the quantity of greenhouse gases that will be emitted in the years to come (period 2000–2100), each RCP scenario gives a variant deemed probable for the climate that will result from the level of emission chosen as working hypothesis. The four scenarios are named after the range of radiative forcing thus obtained for the year 2100: the RCP 2.6 scenario corresponds to a forcing of +2.6 W/m^2^ (Watt per square meter), the RCP 4.5 scenario to +4.5 W/m^2^, and the same for the RCP6 and RCP8.5 scenarios. The latest scenarios, so-called SSPs, are presented according to the efforts that will need to be made in adaptation and mitigation if the world is moving towards such scenarios.

The LMM model is forced by daily rainfall and temperature data from the WFDEI, and the multi-model ensemble mean of the bias-corrected CMIP6 data to develop malaria incidence in different climatological periods, using historical experiments and SSP126, SSP245, and SSP585 emission scenarios separately. The datasets (observed and simulated) used were interpolated on the same grid to make them consistent. The LMM takes climate data at this interpolated resolution. The bias-corrected CMIP6 data used in this work were obtained from the LMDZ server (LMD stands for the Laboratoire de Météorologie Dynamique, the “Z” in LMDZ stands for “zoom” and refers to the regional refinement capacity of the grid). The data processing was performed using a MATLAB language that includes several functions for mapping NetCDF data and organizing time series to be easily used within the box plot function.

The annual cycles of the rainfall, the temperature, and the malaria incidence are estimated from datasets by taking the average of monthly values for all years, and the interannual variability is calculated from datasets by taking the annual mean, that is, the average of all months for a given year. Climatologically, the annual cycle is commonly estimated from datasets by taking the average of all the months of the year. After removing the seasonal cycle first, standardized anomaly values were considered. Standardized anomalies are defined as the difference between annual values and the climatological average; this difference is then divided by the standard deviation of the seasonal values.

Furthermore, the Taylor diagram described by Taylor et al. [20] is used to evaluate the performance of CMIP6 (multi-model ensemble) models against the WFDEI data used as a point of reference. This diagram offers the advantage of representing at the same time three statistical measures, which are the mean squared error (RMSE for root mean square error in English), the standard deviation of the simulation compared to the observation (or reference data), and the correlation coefficient between observation and simulation. It was invented by researcher Karl E. Taylor in 1994 and is frequently used by meteorologists and atmospheric scientists. The most used statistic to quantify the similarity between observed and simulated values is the correlation coefficient. This coefficient makes it possible to detect the presence or absence of a linear relationship between two continuous quantitative characters. The correlation coefficient measures the degree of connection or dependence between two quantitative traits. The best method is the one that maximizes the correlation coefficient. Standard deviation is the most used measure of data dispersion in statistics. The lower the standard deviation, the less the values are dispersed around their mean. The root mean square error is the square root of the arithmetic mean of the square of the difference between the observed and simulated values. The calculation of the RMSE provides us with information on the amplitude of the deviations. The best method is the one that minimizes the RMSE. The RMSE is an appropriate error measure for data that exhibits very high seasonality. The correlation coefficient and the root mean square error provide additional statistical information to quantify the correspondence between two sets of data for a more complete characterization of the observed and estimated data.

### 2.3. LMM Malaria Model

The Liverpool Malaria Model (LMM) is a dynamic model of malaria driven by daily time series of rainfall and temperature. The different components of the malaria transmission model and the calibration of the parameters are described in more detail by Hoshen and Morse [9] then by Ermert et al. [21]. The LMM is a mathematical-biological model of parasite dynamics, which includes an intra-vector phase dependent on meteorological conditions and a phase within the human host independent on meteorological conditions. The mosquito population is simulated using larval and adult stages, the number of eggs deposited in the breeding sites and the larval mortality rate according to the rains of the previous 10 days. The mortality rate of adult mosquitoes and the egg-laying/biting cycle (called the gonotrophic cycle) depend on temperature. The process of parasite transmission between humans and mosquitoes is modeled with a temperature dependence for the parasite reproduction rate (sporogonic cycle) and mosquito biting rate. The two cycles evolve according to the number of “degree-days” above a certain temperature threshold. The gonotrophic cycle takes approximately 37 degree days with a threshold of 9 °C, while the sporogonic cycle takes approximately 111 degree days with a threshold of 18 °C [22]. Climate and health studies have used LMM simulations in southern Africa, including Zimbabwe, Botswana and across the African continent [23,24]. The model’s output variables are, among others, the incidence, prevalence, mosquito population, etc. The current version of the model (LMM2010) [21] has shown significant improvements in the simulation of malaria dynamics in sub-Saharan African countries, including Senegal.

## 3. Results

### 3.1. CMIP6 Models’ Evaluation: Validation of the Rainfall and Temperature Inputs

**Figure 2:**
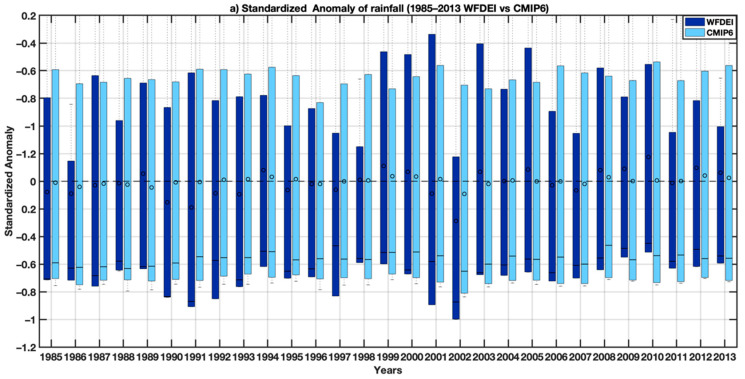

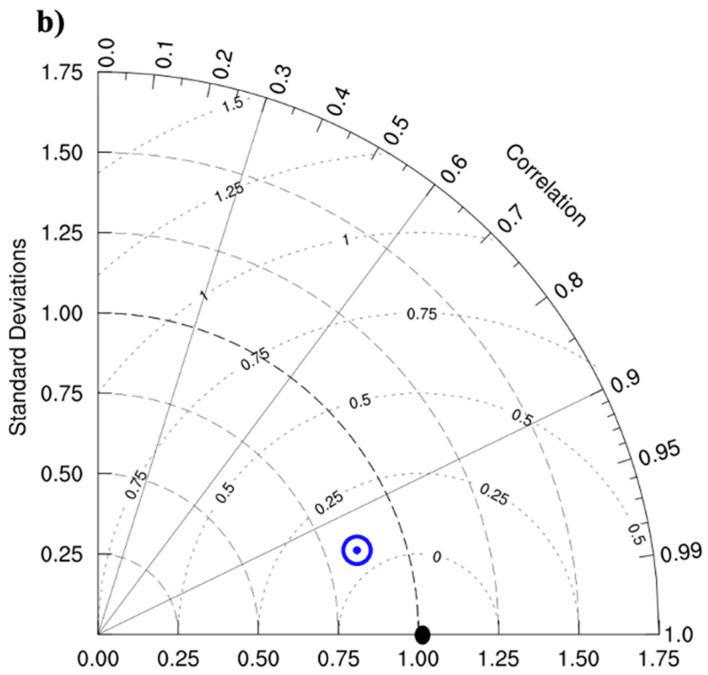
(**a**) Standardized rainfall anomalies showing the interannual variability of rainfall (dark blue bars for the reference data WFDEI and light blue bars for CMIP6 simulations). Boxes indicate the interquartile range and median of the ensemble, whilst the whiskers represent the 5th and 95th percentiles. (**b**) Standardized Taylor diagram displaying the statistics (coefficients, standard deviations and mean square errors) of rainfall comparing multi-model ensemble mean of the bias-corrected CMIP6 with reference climate data (WFDEI). The multi-model ensemble mean of the bias-corrected CMIP6 compared is shown by the blue cycle, and the WFDEI used as a point of reference is shown by the black dot point on the *x*-axis.

Figure 2a shows the distribution of standardized rainfall anomalies for each year of the validation period by comparing historical time series with WFDEI data (observations). The years when the CMIP6 rainfall median is positive (negative) largely correspond to the years when the WFDEI is also positive (negative). In Figure 2b, if we project the axis of the correlations, we note that the correlation coefficient is around 0.95, and for the mean squared error, considering the semicircles whose origin is centered around 1, we see that the blue point is between 0 and 0.25, so approximately, the root mean square error is 0.1. For the standard deviation, considering the semicircles whose origin is centered around 0, we see that the blue point is between 0.75 and 1, so the corresponding value is approximately 0.8.

**Figure 3:**
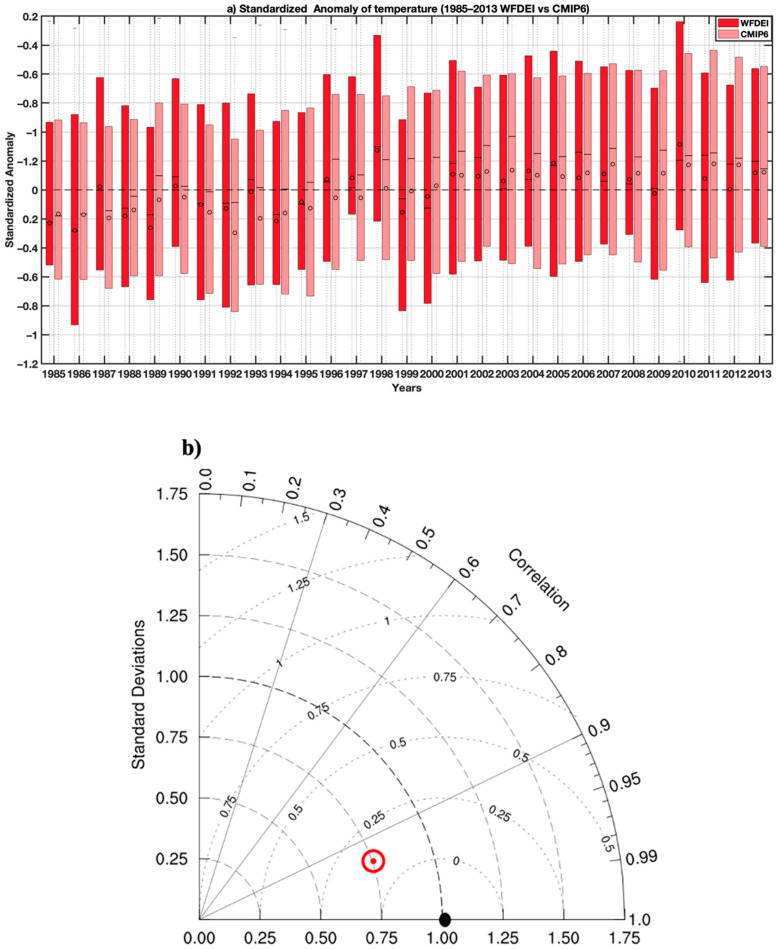
(**a**) Standardized temperature anomalies showing the interannual variability of temperature (dark red bars for the reference data WFDEI and light red bars for CMIP6 simulations). Boxes indicate the interquartile range and median of the ensemble, whilst the whiskers represent the 5th and 95th percentiles. (**b**) Standardized Taylor diagram displaying the statistics (coefficients, standard deviations and mean square errors) of temperature comparing multi-model ensemble mean of the bias-corrected CMIP6 with reference climate data (WFDEI). The multi-model ensemble mean of the bias-corrected CMIP6 compared is shown by the red cycle, and the WFDEI used as a point of reference is shown by the black dot point on the *x*-axis.

Figure 3a shows the distribution of standardized temperature anomalies for each year of the validation period by comparing historical time series with WFDEI data (observations). The years when the CMIP6 temperature median is positive (negative) largely correspond to the years when the WFDEI is also positive (negative) with some exceptions such as for the years 1989 and 1999. In Figure 3b, the correlation coefficient is around 0.93. For the mean squared error, considering the semicircles whose origin is centered around 1, we see that the red point is between 0 and 0.25, so approximately 0.23. For the standard deviation, considering the semicircles whose origin is centered around 0, the corresponding value is exactly 0.75.

### 3.2. Projected Changes in the Spatiotemporal Variability of the CMIP6 Data

#### 3.2.1. Projected Changes in the Spatiotemporal Variability of the CMIP6 Rainfall

**Figure 4:**
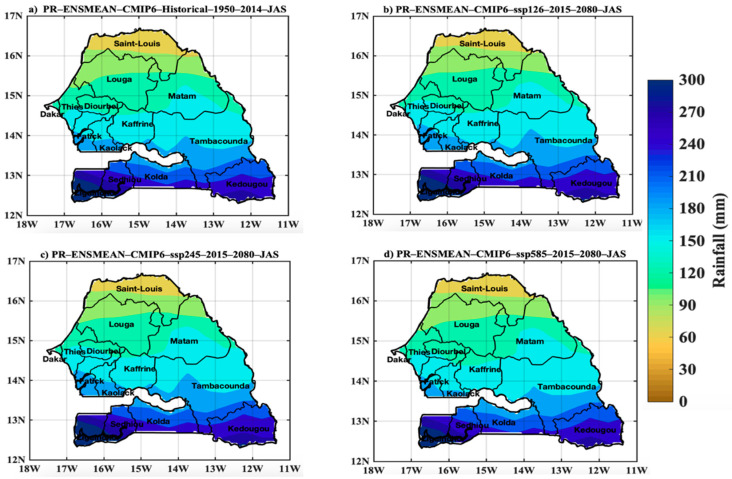
Spatial distribution of rainfall in Senegal in June–August–September with (**a**) historical data (1950–2014), and projections (2015–2080) for the (**b**) scenarios SSP12.6, (**c**) SSP245, and (**d**) SSP585 of the CMIP6 ensemble models. The exact units here are mm per month average.

**Figure 5:**
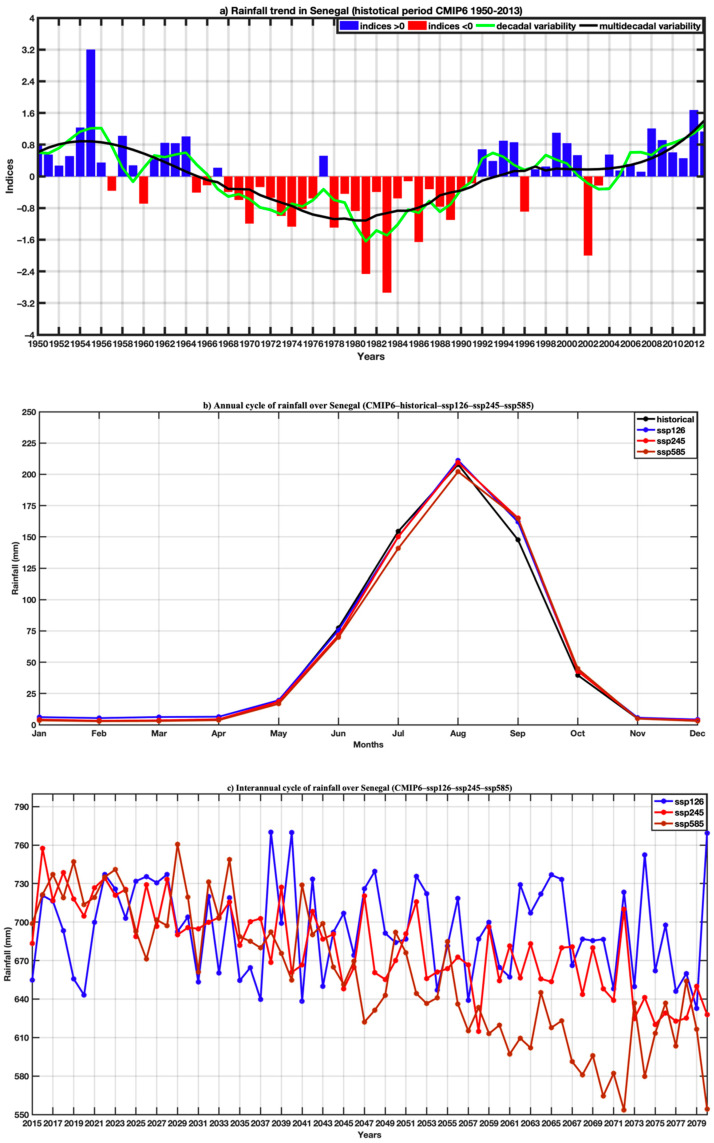
(**a**) Trend of average rainfall over Senegal for the historical period 1950–2013 (overall average of CMIP6 models) highlighting interannual variability, decadal and multi-decadal variability, (**a**,**b**) respectively, annual cycle and interannual variability rainfall in Senegal with historical data (1950–2014), and (**c**) projections (2015–2080) for the SSP126, SSP245, SSP585 scenarios of the CMIP6.

In Figure 4, the spatial distribution of rainfall variables is represented for the historical period (1950–2014), under the SSP126, SSP245 and SSP585 scenarios for the 2015–2080 projection period (common period of the different models used for the ensemble mean). A latitudinal gradient is observed on the rainfall maps. These results also show a decrease in rainfall for the projections. Figure 5a obtained from the ensemble mean of the CMIP6 simulations over the period 1950–2013 exhibits a particular interest explained by the fact that it reveals the largest rainfall deficit ever recorded during the 20th century in Senegal. The marked drought of the 1970s was the most serious event recorded in the historical period. The multi-decadal variability shows an alternation of wet and dry periods. Between two wet periods (1950–1970 and 2005–2010), there was a period of intense drought. The curve representative of the multi-decadal variability shows that during the wet period (1950 and 1960), we observe dry sequences (in 1957, 1960 and 1965, and 1966). Thus, we observe that the year 1977 was relatively wet during the period of the great drought of the 1970s.

In Figure 5b, the variation in the amplitude of rainfall is much more marked between the months of July and August, with the SSP585 scenario. On the other hand, the rainfall regime was almost maintained between the historical and the SSP126 and SSP245 scenarios with a peak of 200 mm in August. The different time series are consistent with the annual cycle characterized by a single rainy season in the year (from May to October) with maximum rainfall in August. Figure 5c represents the interannual variations in rainfall for the historical periods and scenarios SSP125, SSP245, and SSP585. The significant difference between the historical dataset and the three scenarios is noted in the interannual variability (Figure 5c). The comparison of the interannual variability reveals a clear decrease in rainfall for the projections, with the extreme scenario SSP585 and scenarios sSSP126 and SSP245 to a lesser extent.

#### 3.2.2. Projected Changes in the Spatiotemporal Variability of the CMIP6 Temperature

**Figure 6:**
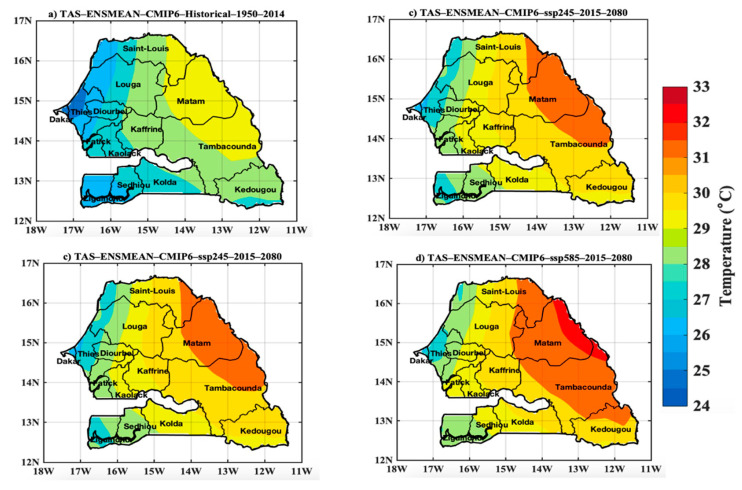
Spatial distribution of temperature in Senegal with (**a**) historical data (1950–2014), and projections (2015–2080) for the (**b**) scenarios SSP12.6, (**c**) SSP245, and (**d**) SSP585 of the CMIP6 ensemble models. The exact units here are °C per month average.

**Figure 7:**
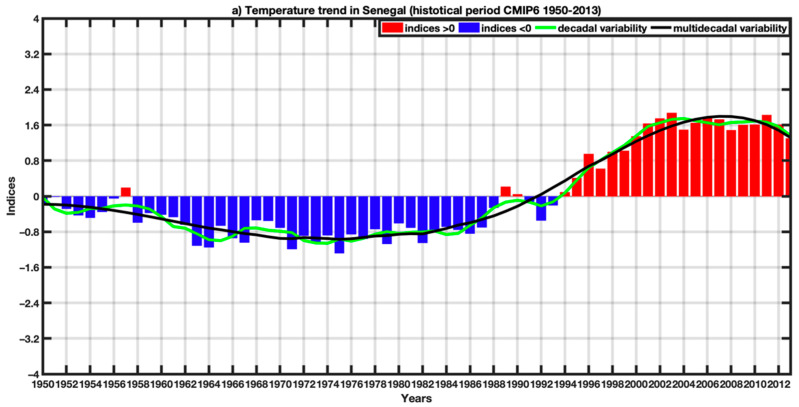

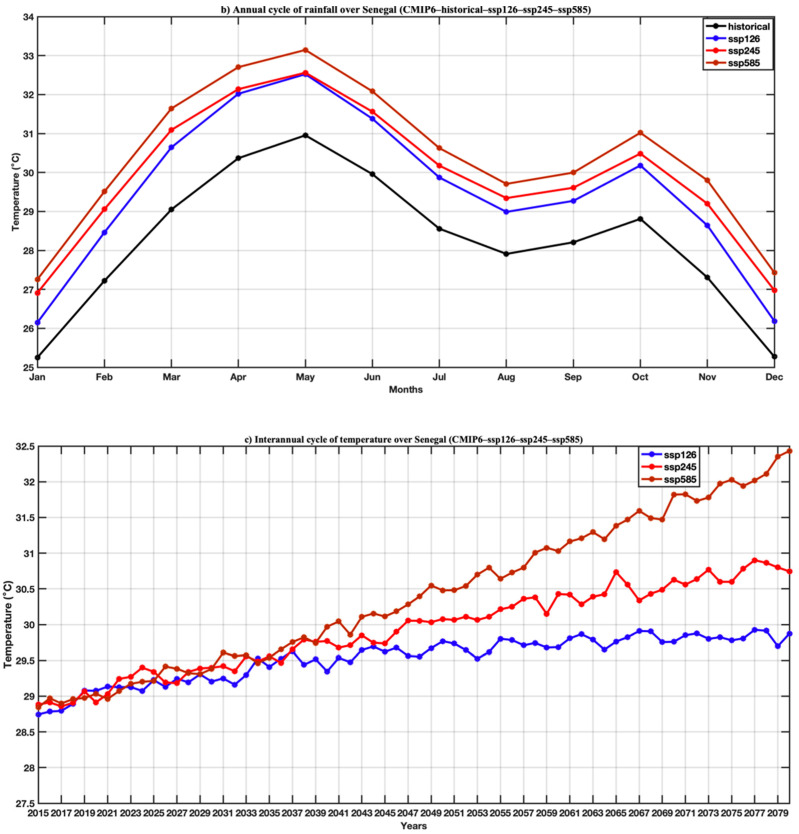
(**a**) Trend of average temperature over Senegal for the historical period 1950–2013 (overall average of CMIP6 models) highlighting interannual variability, decadal and multi-decadal variability, (**a**,**b**) respectively, annual cycle and interannual variability rainfall in Senegal with historical data (1950–2014), and (**c**) projections (2015–2080) for the SSP126, SSP245, SSP585 scenarios of the CMIP.

Figure 6 highlights global warming with an increase in the trend in the projections compared to the historical period. The highest temperature generally prevails in the country’s interior but also in the eastern part of Senegal for historical and projection datasets. The lowest temperature values occur over portions of Senegal’s coastal area. The scenarios SSP245 and SSP585 indicate a sharp increase in temperature for the future, particularly towards eastern parts of Senegal, with monthly average temperature ranges approaching 32 to 33 °C for the SSP245 and 32 °C for the SSP585. Figure 7 represents the evolution of the average temperature over Senegal for the historical period 1950–2013, and the average annual cycles of three time series, namely the historical, and the projections under SSP126, SSP245, and SSP585 scenarios. During the historical period, the upward trend in temperature is better appreciated considering the decadal evolution (Figure 7a). The increase is perceptible over the year-to-year variability, even if the period 1950–1990 is marked by negative standardized anomalies (Figure 7a). The annual regime (Figure 7b) of the temperature with the different time series shows an increase for the projection period, with all three scenarios considered. The annual cycle indicates a bimodal temperature cycle, one in May and the other in October. The analysis of the monthly temperature evolution indicates that the hottest months are March, April, May, June, October, and November with a temperature above 30°C. On the other hand, the temperature is relatively low in August and September with values between 28 and 30 °C due to the influence of cloud cover and heavy rainfall during the rainy season. The temperature remains relatively high during the year. Indeed, the climate conditions during the year in Senegal can be categorized into different seasons:

- between December and January, there is a period marked by a dry climate and very low temperatures linked to the polar invasions during the winter season.
- between February and May, it is very hot and dry with the first peak in temperature in May; this absolute peak in temperature precedes the start of the rainy season;
- the period from July to September (rainy season) is very rainy and wet with mild temperature due to cloud cover;
- the last period of this classification extends between October and November and is marked by high humidity and slightly high temperature. The second peak of the annual temperature cycle often occurs in October with a peak approaching 32 °C.

For interannual variability (Figure 7c), significant changes in temperature are clearly exhibited. We can see a regular trend reflected in rising temperature since the beginning of the projection period (2015). Moreover, we even see this tendency of increased temperature since the historical period, which is getting worse in the projections.

### 3.3. Simulated Malaria Incidence in Senegal

**Figure 8:**
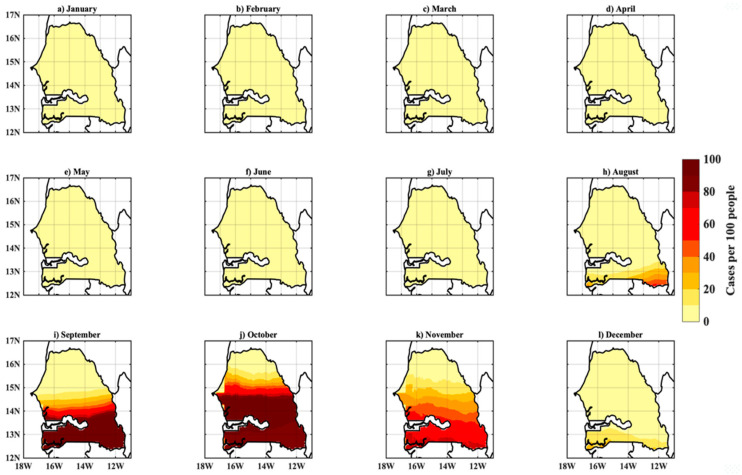
Spatial distribution and monthly evolution of malaria incidence for 1979–2018: LMM model simulations based on WFDEI data. (**a**–**l**) corresponds respectively to the evolution of malaria incidence from January to December over Senegal.

In Figure 8, we analyze the spatiotemporal variability of malaria incidence simulated by the LMM model forced by daily rainfall and temperature data of the reference data (WFDEI). There is a clear difference in the intensity of the malaria incidence signal between the northern and southern regions of Senegal. A very intense signal is observed in the southern part of Senegal, while in the far north, the signal is very weak. Indeed, the strong occurrence only extends around 15° N. Malaria transmission prevails over the country, notably between 11° N and 15° N. A particularly high signal is observed during the September–October–November season with a maximum in October.

**Figure 9:**
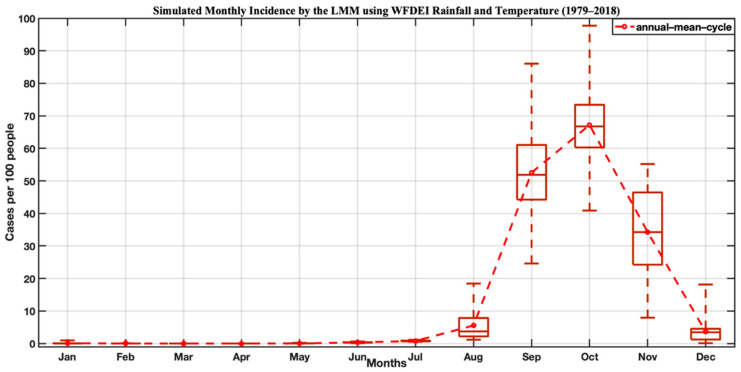
Intra-annual variation in incidence for the period 1979–2018: LMM model simulations based on WFDEI data.

The annual cycle of the simulated malaria incidence is shown in Figure 9. The analysis of the seasonal evolution shows an incidence rate close to zero during the period from January to June. From July, we see an increase in malaria incidence, which peaked in October at 70%. Malaria transmission follows the rainfall regime. The rainy season is a period of high mosquito density. Other studies have shown that the peak of malaria mostly follows the peak of rainfall [6]. Thus, the season of high malaria transmission has a maximum shift of one to two months compared to the rainfall.

**Figure 10:**
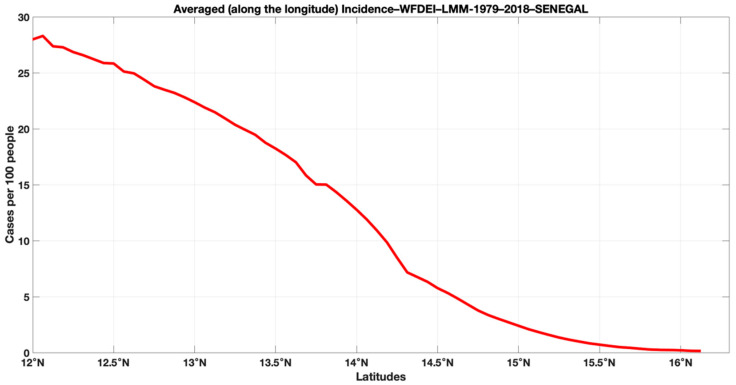
Latitudinal variation in malaria incidence for the period 1979–2018: LMM model simulations based on WFDEI data.

Figure 10 shows the latitudinal malaria incidence gradient, highlighting the difference between the north and the south. We see that the malaria incidence decreases from the latitude 12° N towards the high latitude 16° N. In general, the parameters of malaria increase from north to south. So, they follow the latitudinal gradient of rainfall in Senegal and the environmental conditions.

**Figure 11:**
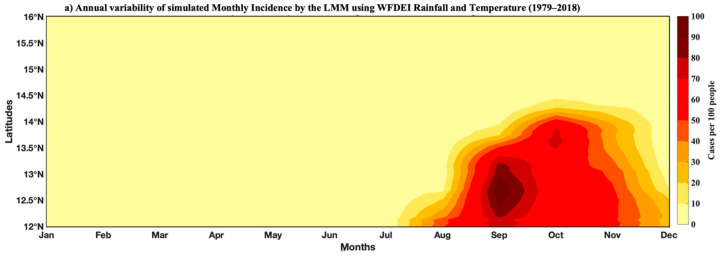

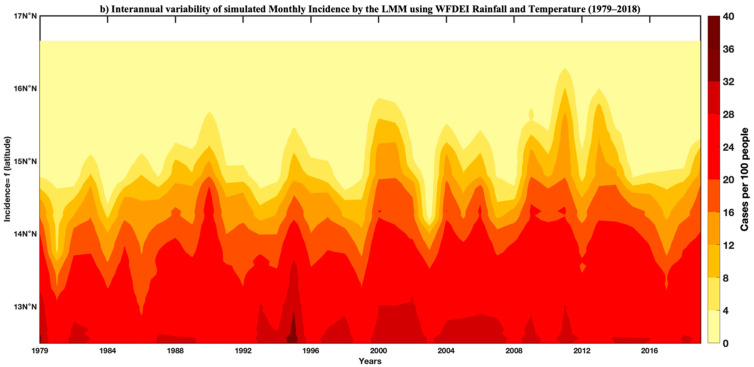
Hovmöller diagram of malaria incidence for the period 1979–2018: LMM model simulations based on WFDEI data: (**a**) annual variability and (**b**) interannual variability.

The Hovmöller diagram (Figure 11) of malaria incidence indicates again that the maximum is observed in September–October. The high malaria transmission season occurs from September to November. High malaria incidence prevails throughout the area with an unequal distribution; the most affected regions are in the south (below 14.5° N) compared to the north (above 14.5° N). This difference between the north and the south is also implied by significant vegetation cover and optimal temperatures for malaria transmission. The Hovmöller diagram also indicates the year-to-year variability of malaria incidence. Figure 11b shows the average intensity of malaria incidence from 1985 to 2014. The signal is particularly strong in some years, including between 2000 and 2016.

### 3.4. Validation of Simulated Malaria Incidence in Senegal

**Figure 12:**
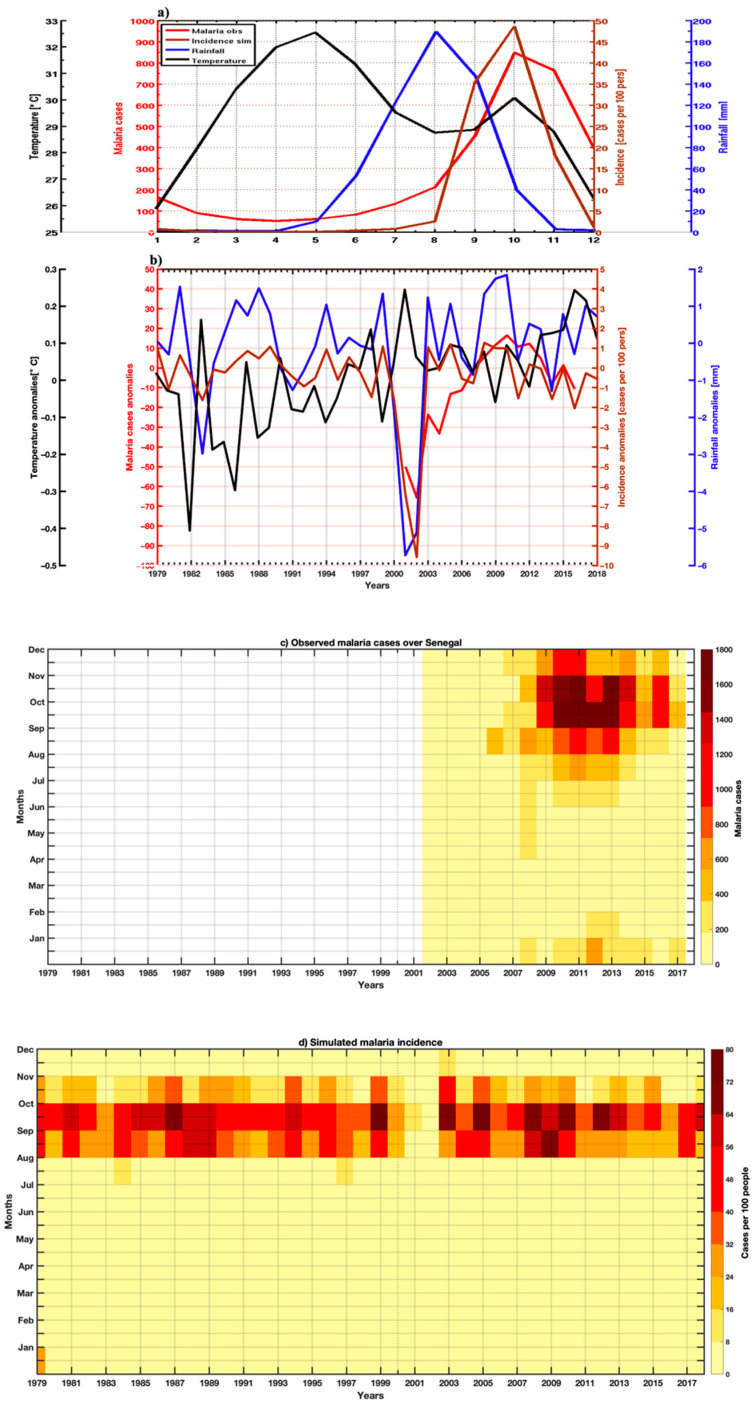
Comparison of the intra- and interannual variability of temperature, rainfall, observed malaria cases, and simulated malaria incidence in Senegal from 2002–2017: (**a**) annual cycles and (**b**) interannual variability; (**c**,**d**) intra- and interannual variability of malaria cases and simulated malaria incidence in Senegal from 2001–2017.

In Figure 12a, the mean annual values of malaria cases, malaria incidence and climatic data (rainfall and temperature) have been presented. The high malaria transmission period extends to three months in observation and simulation. For the observed malaria cases, the period September–November is marked by an increased occurrence of malaria, with the maximum intensity of signal prevailing in October. For the malaria incidence, the substantial transmission period is August–October, with a maximum in October. The model performs well in the simulations of the high malaria transmission period. The interannual variability of the climatic and malaria parameters is shown in Figure 12b. The model well reproduced the interannual variability of transmission. Low malaria in 2002 was implied by particular dryness in Senegal during this year. In general, corresponding years of positive (negative) malaria case anomalies and malaria incidence anomalies during positive (negative) rainfall anomalies are observed. In 2010, 2012, and 2015, high malaria transmission was observed in both observed malaria cases and simulated malaria incidence. A decrease in malaria prevailed in the latest year of the time series. Figure 12b shows that temperature plays a role in the annual variability of malaria. On the other hand, the role that rainfall plays in the transmission of malaria is predominant for a variation from one year to another. In Figure 12c,d, a marked interannual variability of malaria is indicated, and the simulated seasonality of malaria over Senegal is well-represented. A clear corresponding signal with high intensity is found in the observed malaria cases and simulated malaria incidence.

### 3.5. Projected Changes in Malaria Incidence Based on CMIP6 Data

**Figure 13:**
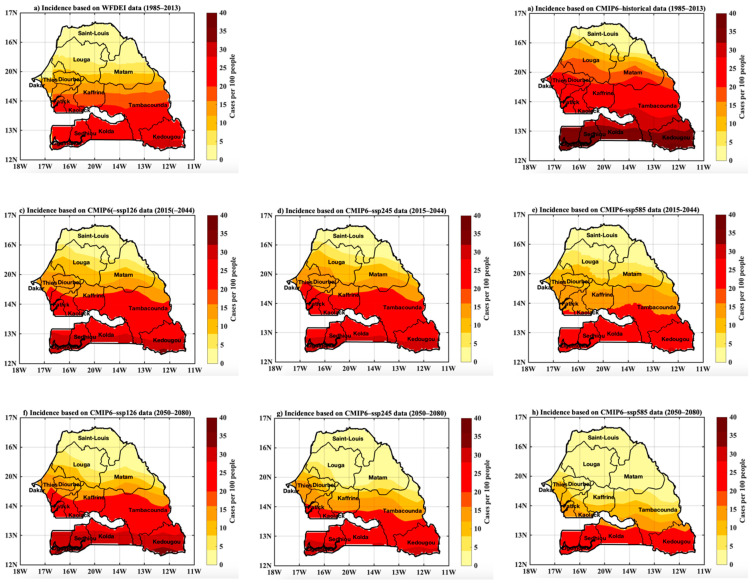
Spatial distribution of malaria incidence: (**a**) for the baseline period 1985–2013 and for simulations based on WFDEI data as reference, (**b**) for the historical period 1985–2013 and for simulations based, respectively, on scenarios SSP126, SSP245, SSP585 of the CMIP6 data, (**c**–**e**) for the near future 2015–2044 and (**f**–**h**) for the far future 2050–2080.

**Figure 14:**
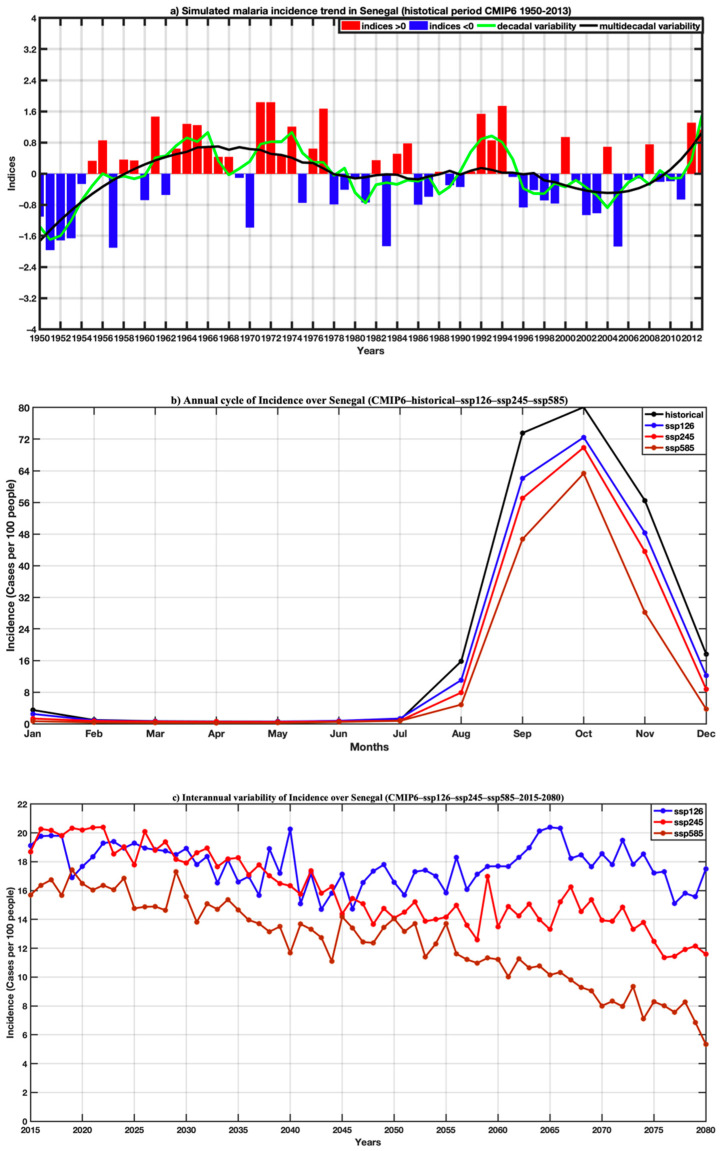
(**a**) Trend in malaria incidence averaged over Senegal for the historical period 1950–2013 (simulation of the LMM model based on precipitation and temperature of the overall average of the CMIP6 models) highlighting the interannual variability, the variability decennial and multi-decadal; (**b**,**c**) respectively, annual cycle of malaria incidence in Senegal with historical data (1950–2013), and projections (2015–2080) for scenarios SSP126, SSP245, SSP585 of CMIP6.

Figure 13a,b illustrate consistency between the malaria incidence simulated by the LMM model forced by the WFDEI (reference data) and that obtained from the CMIP6 data, both with the historical and with the different scenarios. For the spatial variability, there is still a clear difference in the intensity of the signal of the malaria incidence between the northern regions and southern Senegal. The latitudinal gradient of the distribution of malaria in Senegal would be still maintained. By comparing with historical data (Figure 13a,b), we find that the simulated malaria incidence decrease likely tends to prevail over many portions of Senegal, in the north and the center, and this even in the near future (2015–2044), but especially in the far future (2050–2080), with the magnitude of the decrease greater with the SSP245 and SSP585 scenarios (Figure 13d,e,g and Figure 14h). However, looking at the southern part of Senegal in Figure 13c,f and comparing it with Figure 13a as a point of reference, malaria is expected to increase in the southern part of the study area.

Figure 14a illustrates the trend in malaria incidence along the total historical used, i.e., the period from 1950 to 2013. There is a drop, particularly between the 1970s and the 1990s; this decrease would be linked to the decline in rainfall during this period. On the annual cycle in Figure 14b, we observe that malaria transmission increases only between September and November with a peak in October, which is obtained with the climatology of the different time series. The projections in Figure 14c show more clearly the downward trend in malaria incidence, which would be linked to the drop in precipitation mentioned above, but also to temperatures that are too hot for the two scenarios SSP245 and SSP585. As for the SSP126 scenarios, it illustrates a slight increase in malaria incidence in the far future.

## 4. Discussion and Conclusions

Climate projections for the Sahel, particularly in Senegal, include uncertainties for two reasons. On the one hand, because of the strong climate variability observed in the 20th century, it is more difficult to extract from the background noise a signal attributable to climate change; on the other hand, climate models give very divergent results for this region. This divergence is particularly noticeable in the case of rainfall, where even the sign of the change is different between the models. For this reason, we consider the multi-model ensemble mean of CMIP6 data from historical and the projections for rainfall and temperature. Given the wide divergence between models, it is usually advisable not to base assessments of future climate change on the results of a single model taken in isolation; so, in this study, we work on an average of a set of different models of CMIP6 (multi-model ensemble mean). The latitudinal gradient on precipitation in Senegal is well reproduced with CMIP6 data. There is a slight decrease in the precipitation signal in the projections. For the temperature, we note high temperature is more localized in the east of the country but also inside the country to a lesser extent, both for history and for projections. The SSP245 and SSP585 scenarios show a strong increase in temperatures for the future, especially toward eastern Senegal. Three statistical values including the mean squared error, the standard deviation, and the correlation coefficient indicate the performance of CMIP6 in reproducing rainfall and temperature observations (WFDEI). 

The integration of LMM with future climate scenarios reveals the effect of changes in rainfall and temperature on changes in malaria transmission. Based on the findings of this study, malaria is expected to increase in the southern part of the study region in the future. This agrees with earlier findings in previous studies over West Africa that showed epidemic fringe shifted southward for most malaria models. Otherwise, it is expected that by the 2080s, the climate will become unsuitable for malaria vectors in the northern part of the Sahel, with no more additional people at risk. Some studies [25] show an opposing effect of climate change on the global distribution of malaria, and they show a decrease in the simulated malaria behaviors over the Sahel, whatever period and scenario is related to a temperature effect. The ability of the LMM model to simulate the seasonality of malaria incidence at the local scale was assessed. The model reveals a period of high malaria transmission between September and November, with a maximum reached in October. The lag between the peaks of rainfall and malaria incidence is explained by the fact that intermittent rains (or showers) in August can on the one hand strengthen the development of the population of mosquito vectors triggered at the onset of the first rains, but these heavy rains in August mainly flush the female mosquitoes’ eggs deposited on the water surface. In addition, the mild temperatures implied by the succession of rainy days are unfavorable for the mosquitoes’ development from larvae to infectious mature mosquitoes through the pupae stage. On the other hand, with a delay of 1 to 2 months, the mosquito vectors have their living conditions improved with the wastewater, warm and humid conditions [26] (humidity increases the longevity of mosquitoes), and environmental conditions including the availability of ponds and vegetation cover. This delay is quite logical in relation to what is known about the biology of the Anopheles vector and the sporogonic cycle of the plasmodium parasite.

Such a decrease in malaria in the far future appears to be associated with climate change [27]. A decrease in simulated malaria behaviors over the Sahel, regardless of the period and scenario considered is related to temperature effect. Thus, temperatures that are too hot could negatively impact adult mosquitoes’ survival by reducing the population of adult mosquitoes in the model, which implies a decrease in malaria transmission. However, malaria incidence is expected to increase in the southern part of the study area. This agrees with previous studies on West Africa such as Peterson [28] showed that the epidemic fringe would be shifted to the south for most of the malaria models. It is expected that during the 2080s, the climate will become too unsuitable in the northern part of the Sahel, including the northern regions of Senegal, without more people at risk [1].

However, these results must be interpreted with caution as there are still uncertainties related to both the disease model and the CMIP6 projections for the future. Several sources of uncertainties come from the model structural uncertainty. In the far future, the model parameter uncertainty, particularly rainfall, can become relatively more important. This work aimed to ascertain how best to incorporate such a model effectively into a national decision-making process concerning health planning and interventions. Limitations related to uncertainties must be indicated to stakeholders and policymakers. This could be achieved by employing a multi-model experimental setup with additional modeling systems. We will continue in other upcoming projects to improve our understanding of the malaria incidence burdens in Senegal and elsewhere under the threat of climate change.

## Figures and Tables

**Table 1:** List of the global circulation models (GCMs), i.e., biased CMIP6 models used in the study.

Model Name	Institution and Country	Resolution
BCC-CSM2-MR	Beijing Climate Centre (BCC) and China Meteorological Administration (CMA), China	1.1° × 1.1°
CanESM5	Canadian Earth System Model, Canada	2.81° × 2.81°
CESM2	National Centre for Atmospheric Research, Climate and Global Dynamics Laboratory, USA	1.25° × 0.94°
CMCC-CM2-SR5	The Euro-Mediterranean Centre on Climate Change, Italia	2.8° × 1.9°
CNRM-CM6_HR	Centre National de Recherches Météorologiques-Centre Européen de Recherches et de Formation Avancée en Calcul Scientifique, France	0.5° × 0.5°
FGOALS-g3	Flexible Global Ocean-Atmosphere-Land System model Grid-point version 3	2° × 2.3°
GFDL-ESM4	Geophysical Fluid Dynamics Laboratory, USA	1.25° × 1.00°
IITM-ESM	Indian Institute of Tropical Meteorology, India	1.9° × 1.9°
INM-CM5-0	Numerical Mathematics, Russian Academy of Science, Moscow 119991, Russia	2° × 1.5°
IPSL-CM6A-LR	Institut Pierre-Simon Laplace, France	2.5° × 1.3°
MIROC6	Japan Agency for Marine-Earth Science and Technology, Kanagawa 236–0001, Japan	1.4° × 1.4°
MIROC-ES2L	Japan Agency for Marine-Earth Science and Technology, Kanagawa 236–0001, Japan	2.8° × 2.8°
MPI-ESM1-2-HR	Max Planck Institute for Meteorology, High Resolution, Germany	0.9° × 0.9°
NESM3	Nanjing University of Information Science and Technology, Nanjing, China	1.9° × 1.9°
TaiESM	Research Centre for Environmental Changes, Taiwan	1.3° × 1°

## Data Availability

All relevant data are presented within the manuscript. The WFDEI data are fully available without restriction from: ftp://rfdata:forceDATA@ftp.iiasa.ac.at (accessed on 30 October 2022). The bias-corrected CMIP6 data used in this work are available from the LMDz server, but the authors of this review do not have the right to openly make non-open access journal articles public.
